# The analysis of latent fingermarks on polymer banknotes using MALDI-MS

**DOI:** 10.1038/s41598-018-27004-0

**Published:** 2018-06-08

**Authors:** K. Scotcher, R. Bradshaw

**Affiliations:** Biomolecular Science Research Centre, Sheffield Hallam University, Sheffield, S1 1WB UK

## Abstract

In September 2016, the UK adopted a new Bank of England (BoE) £5 polymer banknote, followed by the £10 polymer banknote in September 2017. They are designed to be cleaner, stronger and have increased counterfeit resilience; however, fingermark development can be problematic from the polymer material as various security features and coloured/textured areas have been found to alter the effectiveness of conventional fingermark enhancement techniques (FETs). As fingermarks are one of the most widely used forms of identification in forensic cases, it is important that maximum ridge detail be obtained in order to allow for comparison. This research explores the use of matrix-assisted laser desorption/ionisation mass spectrometry (MALDI-MS) profiling and imaging for the analysis of fingermarks deposited on polymer banknotes. The proposed methodology was able to obtain both physical and chemical information from fingermarks deposited in a range of scenarios including; different note areas, depletion series, aged samples and following conventional FETs. The analysis of forensically important molecular targets within these fingermarks was also explored, focussing specifically on cocaine. The ability of MALDI-MS to provide ridge detail and chemical information highlights the forensic applicability of this technique and potential for the analysis of fingermarks deposited onto this problematic surface.

## Introduction

**Figure 1:**
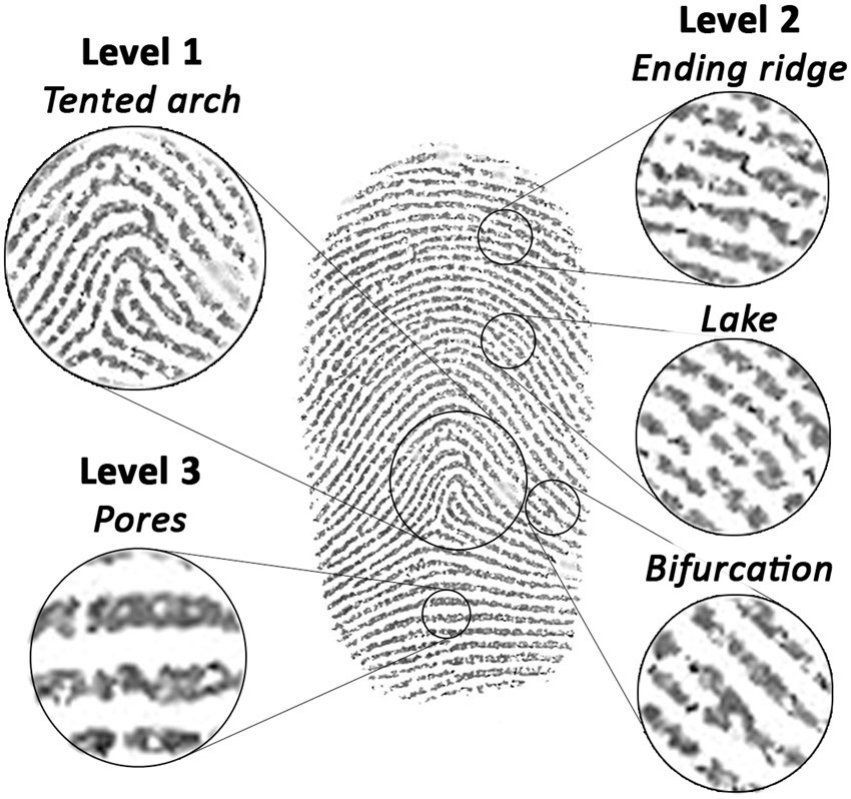
Inked fingermark taken from the primary donor within this research article. Examples of fingermark ridge characteristics have been indicated, including; Level 1 (class of mark - tented arch), Level 2 (*minutiae -* ending ridge, lake and bifurcation) and Level 3 (pores).

Fingermark examination has remained one of the most important and commonly used identification techniques in forensic investigations for over a century^1,2^. It relies on the comparison and matching of ridge detail between a fingermark recovered from an evidential item or crime scene and a control fingerprint stored on record to determine or exclude identification. Ridge detail can be categorized by the class of mark (e.g. loop, whorl and arch) (level 1), small individual characteristics (*minutiae*) (level 2) and fine details (such as the shape and location of pores) (level 3)^2^. A representative example of fingermark characteristics from the primary donor in this research is depicted in Fig. 1.

Latent, or invisible, fingermarks require development in order to observe the characteristics required for identification. There are now a range of fingermark enhancement techniques (FETs) which allow for the development of marks deposited onto a variety of surfaces and subjected to a range of conditions (e.g. wetted marks)^3^. The ‘Fingermark Visualisation Manual’^4^, produced by the UK Home Office, offers a comprehensive overview of the FETs available to practitioners who are involved in the recovery of fingermarks. The manual guides the application of these techniques within systematic workflows, whereby the level of destructiveness increases for each step in order to maintain the integrity of any discovered marks, whilst maximising fingermark recovery.

In September 2016, the UK adopted a new Bank of England (BoE) polymer £5 banknote and the £10 note was introduced in September 2017. Although polymer banknotes were first introduced in Australia in 1988^5^, the transition into UK currency now necessitates the development of appropriate techniques to allow for fingermark recovery. This has been described as of ‘critical importance to the investigation of crime’^6^. The polymeric material, bi-axially oriented polypropylene (BOPP), is a non-porous substrate with several advantages for use within currency, including; increased counterfeiting resilience, cleanliness and strength^7^. However, speciality inks and security features used during the printing and coating process also exhibit semi-porous features^8^ which can affect how the fingermark residue interacts with the surface and subsequently, the FET that can be employed.

Currently in the UK, conventional FETs employed on paper banknotes include DFO (1,8-diazafluoren-9-one) and ninhydrin^4,9^. As these techniques are more suited for porous surfaces, they have proven to be ineffective in developing fingermarks deposited on polymer varieties^6^. Polymer banknotes used in Australia and Canada share the same base material, BOPP, as the BoE £5 polymer banknote^10^. Previous research has demonstrated successful development of fingermarks using vacuum metal deposition (VMD)^11^ and cyanoacrylate fuming (CAF)^12,13^. In particular, it has been observed that there are significant differences in fingermark recovery from specific areas of polymer notes, such as; the transparent security window (whereby CAF was observed to be most effective)^13^ and the opaque sections of the note (whereby VMD processing provided better fingermark development)^6,12,13^. However, differences in inks, opacifying layers, coatings and security features between notes derived from different countries may also have an effect on the success of the conventional FETs^6^.

Recent research on fingermarks deposited onto uncirculated BoE £5 polymer banknotes conducted by Downham *et al*.^6^ showed that multi-metal deposition (MMD) and powder suspensions were among the most effective FETs. It was also determined that CAF and VMD were relatively ineffective, an interesting observation as these techniques are generally considered to be amongst the most effective for marks deposited onto semi-porous and non-porous substrates. This contradicts the results of Jones *et al*.^13^ who reported on the successful application of VMD on Australian polymer banknotes due to the ability of the zinc layer to suppress the multi-coloured and patterned background. Additional techniques which have been used to overcome this issue include IR fluorescence examination^14^ and anti-stokes (“up-converter”) powders^15^.

Over recent years, an area that has gained increasing popularity in fingermark analysis is the use of modern analytical techniques, such as mass spectrometry. These have shown the potential to provide an invaluable insight into the lifestyle of an individual through analysis of the chemical content of their residue^16^, which can also help to identify potential molecular targets for FETs. Furthermore, many of these techniques have the ability to provide additional ridge detail through imaging functionalities which can assist in identification in forensic investigations. A selection of mass spectrometry based methodologies that have been utilised for latent fingermark analysis to-date include; Desorption Electrospray Ionisation Mass Spectrometry (DESI-MS)^17^, Direct Analysis in Real Time Mass Spectrometry (DART-MS)^18,19^, Secondary Ion Mass Spectrometry (SIMS)^20–22^, Surface-Assisted Laser Desorption/Ionisation Mass Spectrometry (SALDI-MS)^19,23–25^ and Laser Desorption Ionisation Mass Spectrometry (LDI-MS)^26^. There has also been an interest in the use of silver sputtering prior to LDI-MS analysis (Ag-LDI-MS), allowing for physical fingermark development and subsequent chemical analysis^27,28^. In fact, Moule *et al*.^28^ recently showed that this technique is useful for a range of porous and semi-porous surfaces (such as polypropylene) but have not yet shown its application to polymer banknotes.

One of the most extensively researched areas within these mass spectrometry based techniques is Matrix-Assisted Laser Desorption/Ionisation-Mass Spectrometry Profiling and Imaging (MALDI-MSP and MSI)^29^. Since the initial publication in 2009, whereby it was shown that a selection of endogenous and exogenous species could be mapped in latent fingermark residue^16^, a wide range of applications for this technology have now been explored^29,30^. Achievements in this area have led to the development of established methodologies for the analysis of fingermarks obtained in real forensic scenarios^31,32^; now allowing for the analysis of fingermarks recovered from crime scenes^33^. The advancements within this niche area of fingermark analysis highlight the power of MALDI-MSP and MSI and the ability to provide additional information from latent fingermarks in forensic scenarios.

The aim of the research presented in this paper was to extend the applicability of MALDI-MS to the analysis of latent fingermarks deposited onto polymer banknotes. In order to prove the feasibility of this technique, the following research questions have been investigated; (a) optimal sample preparation considerations for this surface, (b) the deposition of fingermarks onto different areas (including security features) of the polymer banknotes, (c) the analyses of minute amounts of material in depletion series of both fresh and aged marks, (d) effectiveness when used in sequence with conventional FETs and (e) the detection of forensically interesting substances, namely cocaine. Successful application of this methodology would strengthen the claim that MALDI-MS could be selected as a potential option for fingermark analysis in forensic investigations in the future.

## Materials and Methods

All fingermarks were deposited by two separate donors who provided full consent for use within this study. Ethical approval was granted by Sheffield Hallam University Ethics Research Committee, though the use of fingermarks as samples are considered minor.

### Materials

Trifluoroacetic acid (TFA), α-cyano-4-hydroxycinnamic acid (α-CHCA) and cocaine hydrochloride solution (1 mg/mL in MeOH) were purchased from Sigma Aldrich (Poole, UK). Lab grade ethanol (EtOH), HPLC grade methanol (MeOH), and HPLC grade acetonitrile (ACN) were obtained from Fisher Scientific (Loughborough, UK). Double-sided conductive carbon tape was obtained from TAAB (Aldermaston, U.K.). The materials used for fingermark development; black powder suspension (Wetwop™) and Rite Lok cyanoacrylate superglue, were generously supplied by West Yorkshire Police (Wakefield, UK). BoE £5 polymer banknotes were obtained from a public cash machine (Sheffield, UK). Ultrapure water was obtained from an ELGA Purelab® Ultra water purity system operating at 13.4 MΩ-cm.

### Instrumentation and parameters

MALDI MSI of fingermarks for matrix deposition optimisation (section: Matrix deposition optimisation) was performed using an Applied Biosystems Q-Star XL quadrupole time-of-flight (QTOF) instrument equipped with a Nd:YVO_4_ solid-state laser (Elforlight Ltd., Daventry, UK), having a wavelength of 355 nm, an elliptical spot size of 100 × 150 µm, a pulse duration of 1.5 ns and operating at 1000 Hz.

MALDI-MSP and MSI of all other samples were conducted using a modified Applied Biosystems Q-Star Pulsar *i* hybrid quadrupole time-of-flight (QTOF) instrument, equipped with a Nd:YVO_4_ solid-state laser (Elforlight Ltd., Daventry, UK), with a wavelength of 355 nm and a pulse duration of 1.5 ns, operating at 5000 Hz. All images were acquired at a spatial resolution of 150 × 150 µm using a ‘slow’ raster mode with ‘oMALDI Server 5.1’ software supplied by MDS Sciex (Concord, Ontario, Canada). The elliptical spot size of the laser was 100 × 150 µm. The mass range analysed was between *m/z* 50–1000. The declustering potential 2 was set at 15 and the focusing potential at 20.

MALDI-MSP spectra were obtained in positive ion mode within the mass range of *m/z* 50 and 1000. The declustering potential 2 was set at 15 arbitrary units and the focus potential at 20 arbitrary units. MALDI-MS/MS spectra were produced on the same instrument in positive ion mode as a product ion scan for cocaine (*m/z* 304.15). The mass range was set between *m/z* 50 and 310 with the declustering potential 2 set at 15, the focusing potential at 20, the collision gas at 3 and the collision energy at 25 arbitrary units.

Optical images of all fingermarks were taken using a Foster and Freeman video spectral comparator (VSC 4CX) under incandescent light.

### Data processing

MALDI-MS and MS/MS profiling spectra were processed using the multifunctional open source mass spectrometry software, mMass^34^. All MALDI-MS images were processed using Biomap (Novartis, Basel). In all instances, MALDI-MS imaging data was manually interrogated in order to recover the most abundant ions (providing the best ridge clarity) derived from each separate fingermark analysis, which were then normalised against the total ion current (TIC). The ridge pattern continuity and clarity was used to determine the quality of the fingermark according to the grading scheme published by Bandey (2004)^35^ (Table 1).

**Table 1:** Grading scale for fingermark quality^35^.

Score	Level of detail
0	No development
1	Evidence of contact but <1/3 of mark continuous ridges
2	1/3–2/3 of mark continuous ridges
3	>2/3 of mark continuous ridges but not quite a perfect mark
4	Full development – whole mark continuous ridges

### Banknote cleaning

As bank notes were obtained from a public cash machine, a protocol was optimised (data not shown) to remove any contamination from the note surface and to ensure reproducibility between samples. In each instance, the notes were soaked in EtOH for 5 min before being rinsed with ultrapure H_2_O for 2 min and left to dry in ambient conditions on clean tissue paper.

### Fingermark deposition

Ungroomed fingermarks were prepared by washing the hands with a 50:50 EtOH:H_2_O solution before conducting 15 minutes of normal working activities on a computer which had been cleaned with the same solution. The fingertips were rubbed together thoroughly prior to fingermark deposition to ensure an even distribution of fingermark constituents on the fingertips. All fingermarks were deposited by the same donor (**donor 1**) throughout the research unless stated otherwise. The use of ungroomed fingermarks was based on Home Office recommendations^36^ to avoid the use of purposely ‘loaded’ fingermarks in order to test the feasibility of a method and to avoid the extensive variation that may be observed in natural marks.

### Matrix deposition optimisation

In ***Experiment A***, an ungroomed fingermark was deposited onto the opaque section of a BoE £5 polymer banknote before being cut into quarters. Each quarter was separately sprayed with 2, 3, 4 and 5 layers, respectively, using 5 mg/mL α-CHCA in 70:30 ACN:0.5% TFA. A flow rate of 2 µl/min on a ‘slow’ raster setting was employed using a SunCollect sample preparation device or ‘auto-sprayer’ (SunchromGmbH, Friedrichsdorf, Germany), adapted from previous methodology^16^. The quarters were then attached to a MALDI OPTI spotless insert (Applied Biosystems, CA, U.S.A.) in their original orientation using double-sided conductive carbon tape before being analysed using MALDI-MSI.

In ***Experiment B***, a different donor (**donor 2**) deposited an ungroomed fingermark onto the same opaque section of a £5 polymer banknote which was subsequently split into two halves. One section was coated with 2 layers and the other with 4 layers of MALDI matrix using the same methodology described in ***Experiment A***.

The optimised conditions were determined to be 2 layers of 5 mg/mL α-CHCA MALDI matrix in 70:30 ACN:0.5% TFA, each at a rate of 2 µl/min on a ‘slow’ raster setting using the ‘SunCollect’ auto-sprayer. These spraying parameters were were employed for all subsequent experiments.

### Depletion series and aged marks

A depletion series of eight fingermarks from an ungroomed fingertip were deposited on the opaque section of a £5 polymer banknote. The 1^st,^ 3^rd^, 5^th^ and 8^th^ marks in the series were cut from the polymer banknote, secured on a MALDI target plate and spray coated with the MALDI matrix before being subjected to MALDI-MSI analysis.

In an additional experiment, a depletion series of five fingermarks from an ungroomed fingertip were deposited onto the opaque section of a £5 polymer banknote and left to age in a wallet for 6 weeks. The wallet was largely stored within the owner’s back pocket throughout the day. Although this experiment was intended to replicate realistic conditions, in order to avoid external contamination, the note was stored within a paper envelope. Following the specified ageing period, the marks were cut directly from the note, secured on a MALDI target plate using double-sided carbon tape and spray coated with matrix before being analysed using MALDI-MSI.

### Fingermarks deposited on different areas of a polymer banknote

Ungroomed fingermarks were deposited onto various areas of a £5 polymer banknote, namely; the transparent window, security foil, raised print and Blenheim maze areas^7^. The areas were cut from the note, secured directly on a MALDI target plate using double-sided carbon tape and spray coated with matrix before being submitted to MALDI-MSI analyses.

### Fingermark enhancement and MALDI-MSI analysis

An ungroomed fingermark was deposited on the opaque area of a £5 polymer banknote. The fingermark was cut into quarters and each quarter was subjected to a different enhancement protocol; (A) no enhancement, (B) Cyanoacrylate fuming (CAF) followed by black Wetwop^TM^ powder suspension (C) CAF and (D) Black Wetwop^TM^ powder suspension. The Wetwop^TM^ powder suspension was employed as per the Home Office guidelines^37^.

CAF was performed in a FOLD-A-LAB chamber (Lynn Peavey Company). Following method optimisation (data not shown), a protocol was developed which required a total of 2.5 g of superglue and a beaker containing 100 mL of water being placed onto a hot plate. The section of note containing a fingermark was hung within the chamber and left for 40 min to develop.

Following development using the specified methodologies, the quarters were arranged together into the original orientation, attached to a MALDI target plate using double-sided carbon tape and spray coated with matrix before being imaged using MALDI-MSI.

### Profiling cocaine and imaging cocaine contaminated marks

In an initial experiment, a dilution series of cocaine hydrochloride was prepared in MeOH between 0.1 ng/mL–10 µg/mL. A total of 0.5 µL of each solution was deposited, in triplicate, directly onto a polymer banknote surface and left to dry. 0.5 µL of the MALDI matrix solution was then spotted directly onto each of the cocaine spots before being subjected to MALDI-MS and MALDI-MS/MS profiling. All analyses were conducted in ascending concentrations to avoid carry-over and blanks were analysed between samples.

The three lowest concentrations (10 ng/mL, 0.1 µg/mL and 1 µg/mL) detectable within the first experiment were used to contaminate three separate fingermarks using a modification of the contact-transfer methodology previously described in Groeneveld *et al*.^38^. In this method, a total of 100 µl of each cocaine solution was applied to a clean Petri dish. An ungroomed fingertip was rubbed in the cocaine solution and was allowed to dry before being deposited onto a polymer banknote. Following fingermark deposition, 0.5 µL spots of the MALDI matrix were deposited in various areas of the residue before being subjected to MALDI-MS and MALDI-MS/MS profiling.

An ungroomed fingermark was then prepared following contamination with 100 µL of the lowest cocaine concentration detectable (0.1 µg/mL) using the previously described contact-transfer methodology (total of 10 ng cocaine). This mark was cut from the note and secured directly on a MALDI target plate using double-sided carbon tape before being spray coated with MALDI matrix and imaged using MALDI-MSI. Following MALDI-MSI analysis, the same sample was then subjected to MALDI-MS/MS analysis to confirm the presence of cocaine.

In all instances, a ‘blank’ region of the note was also analysed using MALDI MS and MS/MS in order to confirm that the illicit substance was not present on the note surface prior to fingermark deposition.

## Results and Discussion

### Matrix layer optimisation

**Figure 2:**
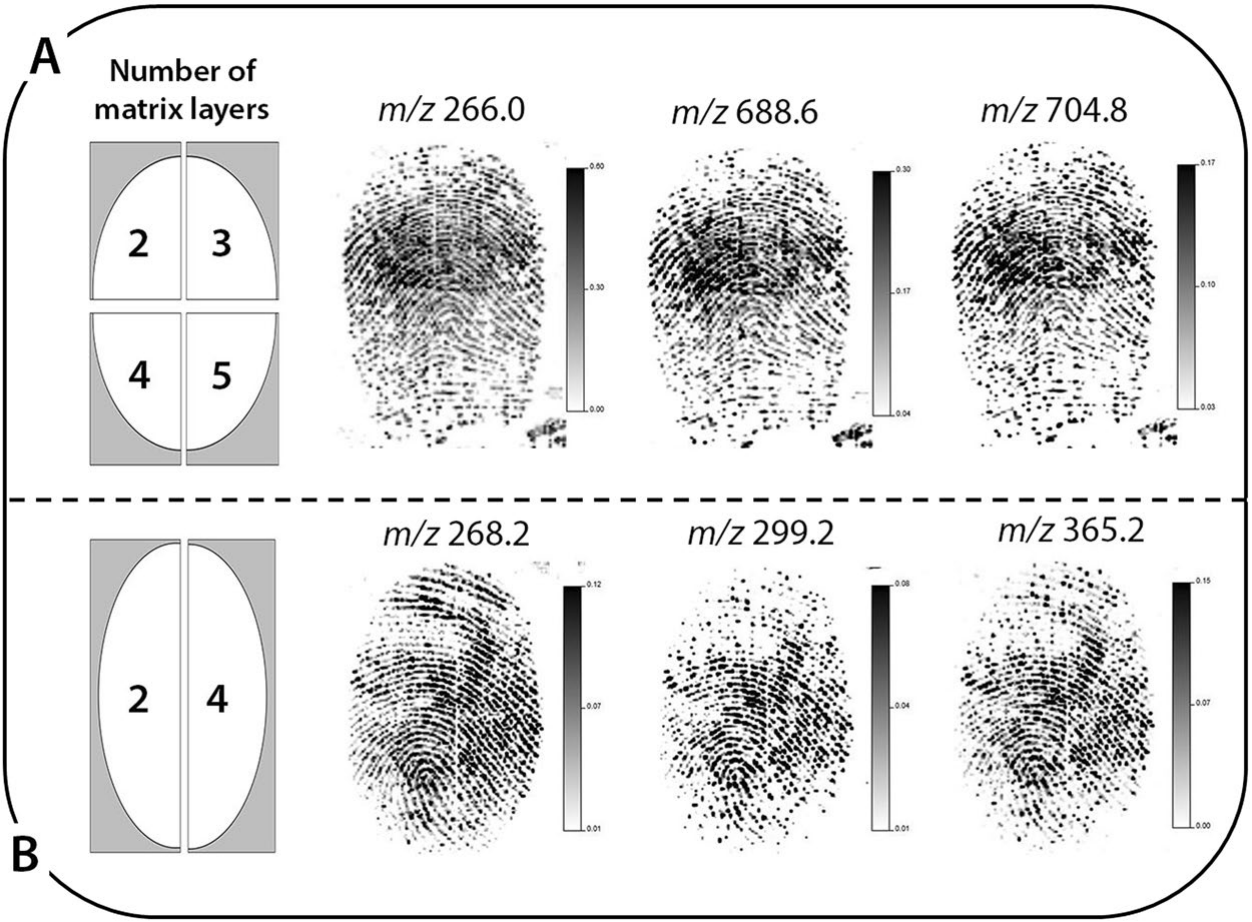
Optimisation of matrix deposition for fingermarks of two donors deposited onto polymer banknotes, including; (**A**) the number of matrix layers sprayed onto four quarters of fingermark 1 (donor 1) and subsequent MALDI MS images acquired, and (**B**) the number of matrix layers sprayed onto two halves of fingermark 2 (donor 2) and subsequent MALDI MS images acquired. In both cases, the quality of ridge detail is the same across each section of the marks, suggesting a 2-layer approach as the most suitable methodology.

In an initial experiment (***Experiment A***), an ungroomed fingermark was deposited onto the opaque area of a polymer banknote and was split into quarters. Each quarter was sprayed with a different number of layers of the MALDI matrix before being subjected to MALDI-MSI analysis. Three different unidentified ion species (*m/z* 266.0, *m/z* 688.6 and *m/z* 704.8) were selected for fingermark ridge reconstruction, which showed no difference in the quality of the fingermark ridge detail in each of the quarters (all grade 3) (Fig. 2A).

To corroborate the initial findings from ***Experiment A***, a second experiment (***Experiment B***), was conducted whereby a second donor (**donor 2**) deposited an ungroomed fingermark onto the same area of a note. This mark was split into two halves before being sprayed with 2 and 4 layers of the MALDI matrix, respectively. Similarly to the results obtained in ***Experiment A***, the two halves of the fingermark showed similar quality of ridge detail (grade 3) for each of the ion species selected for fingermark reconstruction (*m/z* 268.2, *m/z* 299.2 and *m/z* 365.2) (Fig. 2B). Although the ion species at m/z 268.2 remains to be unidentified^31^, putative identifications have been assigned to *m/z* 299.2 (nonadecanoic acid) [M + H]^+^^31^ and *m/z* 365.2 (tetracosadienoic acid) [M + H]^+ ^^39^, which have both been observed within latent fingermark residue previously. Tandem mass spectrometry analysis would be required to confirm the identity of these species.

Based on the MALDI-MS imaging results, the 2-layer methodology was selected for all future sample preparation. This improves upon previous research, whereby fingermark samples have been coated with either four^32,33^ or five^31,40^ layers of MALDI matrix. Not only would this reduce sample preparation time, but it also has the potential to improve spray-coating reproducibility. Analysing the fingermarks of multiple donors also addresses an important issue, highlighted by Ferguson *et al*.^39^, who had shown that results vary across different individuals. The donors in the aforementioned study were subsequently labelled as being ‘good’, ‘intermediate’ or ‘poor’ secretors in respect to the results obtained. The consistent results observed for fingermarks deposited onto polymer banknotes, in this instance, provides a positive indication of reproducibility of the 2-layer methodology which is an important factor when considering forensic applicability. It must be noted however, that additional experiments are still required on a greater number of donors and natural marks to fully assess the reproducibility of this method.

The use of ungroomed fingermarks throughout this research not only ensures that the minimal amount of endogenous and exogenous components were present (highlighting the sensitivity of the technique), but they also allow for a greater amount of reproducibility to be maintained over extended periods of time in comparison to natural marks. Previous research regarding the enhancement of latent fingermarks deposited on polymer banknotes has been conducted using natural, groomed or eccrine fingermarks^6,11,41^ which will generally contain a greater amount of variation and larger quantity of constituents relative to ungroomed fingermarks.

### Depletion series

**Figure 3:**
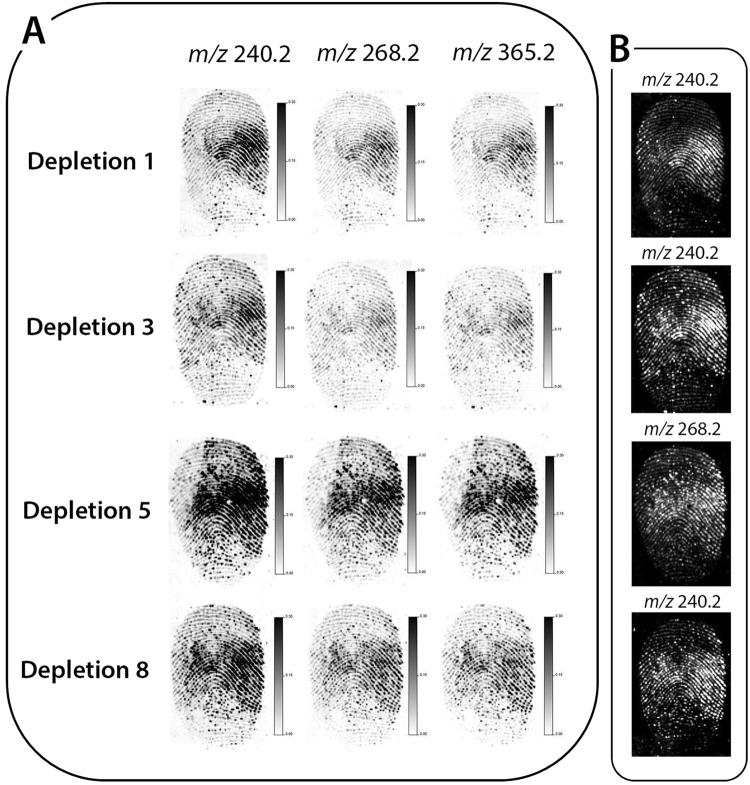
MALDI MS images of a fingermark depletion series (1^st^, 3^rd^, 5^th^ and 8^th^ depletions). (**A**) MALDI MS images of 3 ions, including; two unidentified species (*m/z* 240.2 and *m/z* 268.2) and protonated tetracosadienoic acid (*m/z* 365.2). The images were set to the same contrast and intensity, showing no decrease in ion signal or ridge detail clarity across the 8 depletions and (**B**) MALDI MS images of the ion species providing the best ridge detail for each fingermark, showing that Grade 2 fingermark quality could be obtained in each instance.

A depletion series of eight ungroomed fingermarks was deposited onto the opaque section of a polymer banknote. The 1^st^, 3^rd^, 5^th^ and 8^th^ depositions were selected and subjected to MALDI-MSI analysis. Three ion species were selected to generate molecular images for all depletions, including; unidentified ion species at *m/z* 240.2 and *m/z* 268.2 and tetracosadienoic acid (*m/z* 365.2) [M + H]^+^. By normalising all images and setting them to the same contrast and intensity, it showed that there was no reduction in the intensity of the ion species throughout the depletion series (Fig. 3A). Furthermore, selection of the molecular image with maximum ridge detail and optimal contrast and intensity indicated that the overall quality of fingermark ridge detail recoverable from each of the depletions was consistent (all Grade 2) (Fig. 3B).

The investigation of depletion series has importance in forensic applicability, as individuals will touch surfaces multiple times and limited residue may be present in the fingermark analysed. Further depletions could be explored in future studies to assess the minimal amount of material that can be detected using MALDI-MSI.

### Different areas of a polymer banknote

Fingermarks were deposited onto different areas of the £5 polymer banknote in order to determine whether the MALDI-MS image quality differed depending on differences in the composition of the surface. Specific focus was on the security features, including; the raised print, security foil, Blenheim maze and transparent window. The interaction with the fingermark residue may be affected by the different areas of this complex surface, and will ultimately affect the FET selected. Previous research on Australian and Canadian polymer banknotes showed large variation in visualisation of marks deposited on the opaque and transparent sections^13,42^.

**Figure 4:**
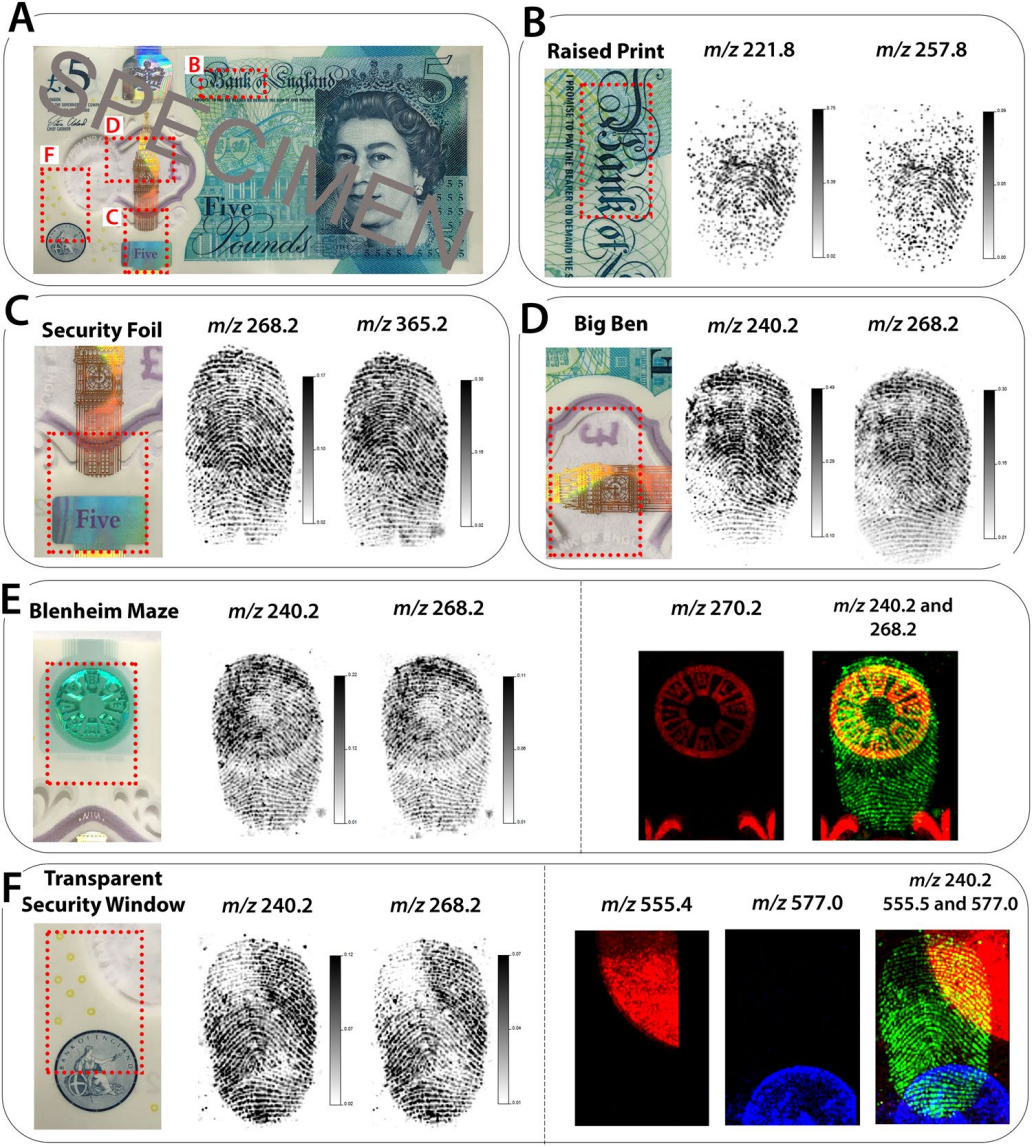
MALDI MSI of fingermarks deposited onto different areas of a polymer banknote. (**A**) An optical image of the Queen side of the banknote and the different areas of the note that were analysed. (**B**–**F**) Zoom-in of each of the areas where fingermarks were deposited and subsequent MALDI MS images acquired from these regions. The areas include; (**B**) raised print, (**C**) security foil and transparent window, (**D**) Big Ben transparent window, (**E**) Blenheim maze and (**F**) transparent security window. In Panels (E,F), overlapping images have been included to highlight the strong signal obtained from the background security features.

MALDI-MSI analysis of fingermarks deposited on to the different areas showed that both physical and chemical information could be observed in each instance. In fact, grade 2 images were obtained for fingermarks deposited onto the raised print area (*m/z* 221.8 and *m/z* 257.8, both unidentified) and grade 3 on the security foil and Big Ben transparent window areas (Fig. 4B–D, respectively). The Blenheim maze region of the polymer banknote exhibited a strong signal for this security feature at *m/z* 270.2 (unidentified) which resulted in signal suppression in the corresponding areas of the fingermark deposited onto this region of the note, shown through the selection of two unidentified species (*m/z* 240.2 and *m/z* 268.2, respectively) (Fig. 4E). Signal suppression is a known issue in MALDI-MS and the effect can arise from contaminants in the sample, on the substrate or the physicochemical properties of the matrix^43^. Nonetheless, it is important to note that significant ridge detail could still be obtained from the areas affected by signal suppression, and Grade 3 fingermark images were obtained overall.

Interestingly, for the fingermark deposited onto the transparent window security feature, strong signals were obtained for two unidentified species distributed within both the transparent window and the Bank of England stamp (*m/z* 555.4 and *m/z* 577.0, respectively). However, images generated for two unidentified fingermark constituents (*m/z* 240.2 and *m/z* 268.2, respectively) revealed that this did not affect the ability to obtain chemical and physical information from the fingermark deposited onto these regions (Fig. 4F). In fact, poorer quality of ridge detail was observed in the regions of the fingermark that were not deposited onto the security features. Although the signal intensity in these regions appeared to be reduced, the overall quality of the fingermarks was still determined to be Grade 3.

### Fingermark enhancement and MALDI-MSI analysis

Although these initial results highlighted the potential of employing MALDI-MSI as a means to analyse fingermarks deposited onto polymer banknotes, this methodology would be redundant without initial development using appropriate FETs. This is necessary to indicate the presence or location of a fingermark prior to MALDI-MS analyses^31–33,38,40^. FETs successfully employed on polymer banknotes include MMD, power suspension^6^, VMD^11^ and CAF^12,13^. However, in this research, CAF and black powder suspension (Wetwop^TM^) were selected as the most appropriate FETs due to equipment availability.

**Figure 5:**
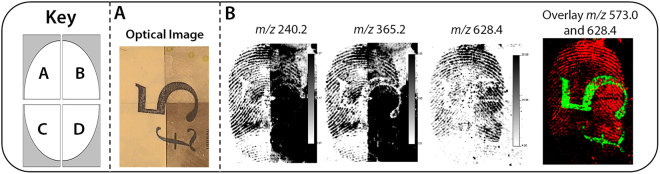
MALDI MSI analysis of a latent ungroomed fingermark deposited onto a polymer banknote following development using conventional FETs. The key indicates the FET used on each section (**A)** - No enhancement, (**B**) CAF followed by black powder suspension (Wetwop^TM^), (**C**) CAF, (**D**) (Wetwop^TM^). Panel (A) shows an optical image following enhancement of the fingermark, whereby ridge detail was only observed in section (**B**). Panel (B) shows the subsequent MALDI MS images, indicating an improvement in ridge detail for sections (**B**,**C)** when selecting an unidentified species and tetracosadienoic acid [M + H]^+^ (*m/z* 240.2 and *m/z* 365.2) and full fingermark reconstruction when selecting an unidentified species (*m/z* 628.4). The overlapping image depicts a strong background signal obtained from the ‘£5’ security feature.

In order to assess compatibility, an ungroomed fingermark was deposited onto a polymer banknote and was split into quarters. Each quarter was subjected to different enhancement methodologies, namely; (A) no enhancement, (B) CAF followed by Wetwop^TM^ in a sequential workflow, (C) CAF and (D) black powder suspension (WetWop^TM^). Following conventional enhancement, ridge detail was only observed within quarter (B), though this was through negative enhancement due to background staining (Grade 1). No fingermark ridge detail was observed in the undeveloped, CAF enhanced sections, and black Wetwop^TM^ powder suspension caused background staining on the polymer banknote, with no ridge detail being observed (all grade 0) (Fig. 5A).

These results are in disagreement with the work of Downham *et al*.^6^ who had demonstrated that powder suspensions show the greatest potential for fingermark visualisation on BoE £5 polymer banknotes (in addition to MMD). The poor results, therefore, could be due to the hypothesised mechanism for powder suspensions, whereby it is assumed that water insoluble fatty components within a fingermark destabilise the detergent causing the powder to interact with the deposited fingermark^37^. As ungroomed fingermarks were employed in this study (containing a minimal amount of fingermark material), the effectiveness of the FET will have been reduced in comparison to these previous studies which used natural and groomed marks.

The results obtained from CAF developed marks, however, are in agreement with prior research on Australian, Canadian and the new £5 polymer banknotes which found poor visualisation of ridge detail after CAF enhancement when compared to other techniques^6,13,42^.

Post-enhancement MALDI-MSI analysis showed that clear ridge detail could be observed at multiple *m/z* species for the undeveloped and cyanoacrylate fumed sections of the deposited fingermark including an unidentified species and tetracosadienoic acid [M + H]^+^ (*m/z* 240.2 and *m/z* 365.2). Furthermore, molecular imaging of an unidentified fingermark constituent at *m/z* 628.4 allowed for ridge detail to be observed in all four sections (Grade 2). In each instance, the background signal of the banknote affected image quality, with a number “5” in the centre of each fingermark. An intense signal was observed for an unidentified species within this region (*m/z* 573.0), as shown within an overlay image (Fig. 5B), providing further indication that certain banknote features can cause signal suppression.

The improvement in the clarity of physical characteristics and generation of chemical information within CAF enhanced fingermarks corroborates the results obtained in Bradshaw *et al*.^31^. However, this is in disagreement with the work of Groenveld *et al*.^38^, who found undeveloped marks to have better ridge detail than CAF enhanced marks when subjected to MALDI-MSI, though in these instances marks were deposited onto an aluminium substrate. These results demonstrate that if a FET does not provide sufficient ridge detail, but has indicated the presence and location of a fingermark, MALDI-MSI can be used to further enhance the ridge detail and provide additional chemical intelligence about the donor.

As the enhancement techniques used in this research did not provide sufficient development of deposited fingermarks, further research into the most effective enhancement techniques for fingermarks on polymer banknotes in conjunction with MALDI-MSI should be conducted. For example, metal deposition (VMD and MMD) has been referenced amongst the most effective fingermark enhancement techniques on polymer banknotes^6,11,12^. VMD has previously been used to enhance fingermarks prior to MALDI-MSI on different surfaces, where it was shown that this technique enhances the signal obtained via subsequent MALDI-MS analyses^31,38^. Therefore, in future work, it would be interesting to observe the effect of this workflow on marks deposited onto polymer banknotes.

### Aged fingermarks

In order to mimic a realistic forensic scenario, a depletion series of 5 ungroomed fingermarks were deposited on a polymer banknote and left to age in a wallet for 6 weeks. Throughout this period, the wallet was used as normal, being stored in the users back pocket between uses. The storage conditions of the aged fingermarks would have subjected the banknote to higher temperatures and pressures than if simply aged at room temperature; however the exact pressure and temperatures could not be monitored during this time.

**Figure 6:**
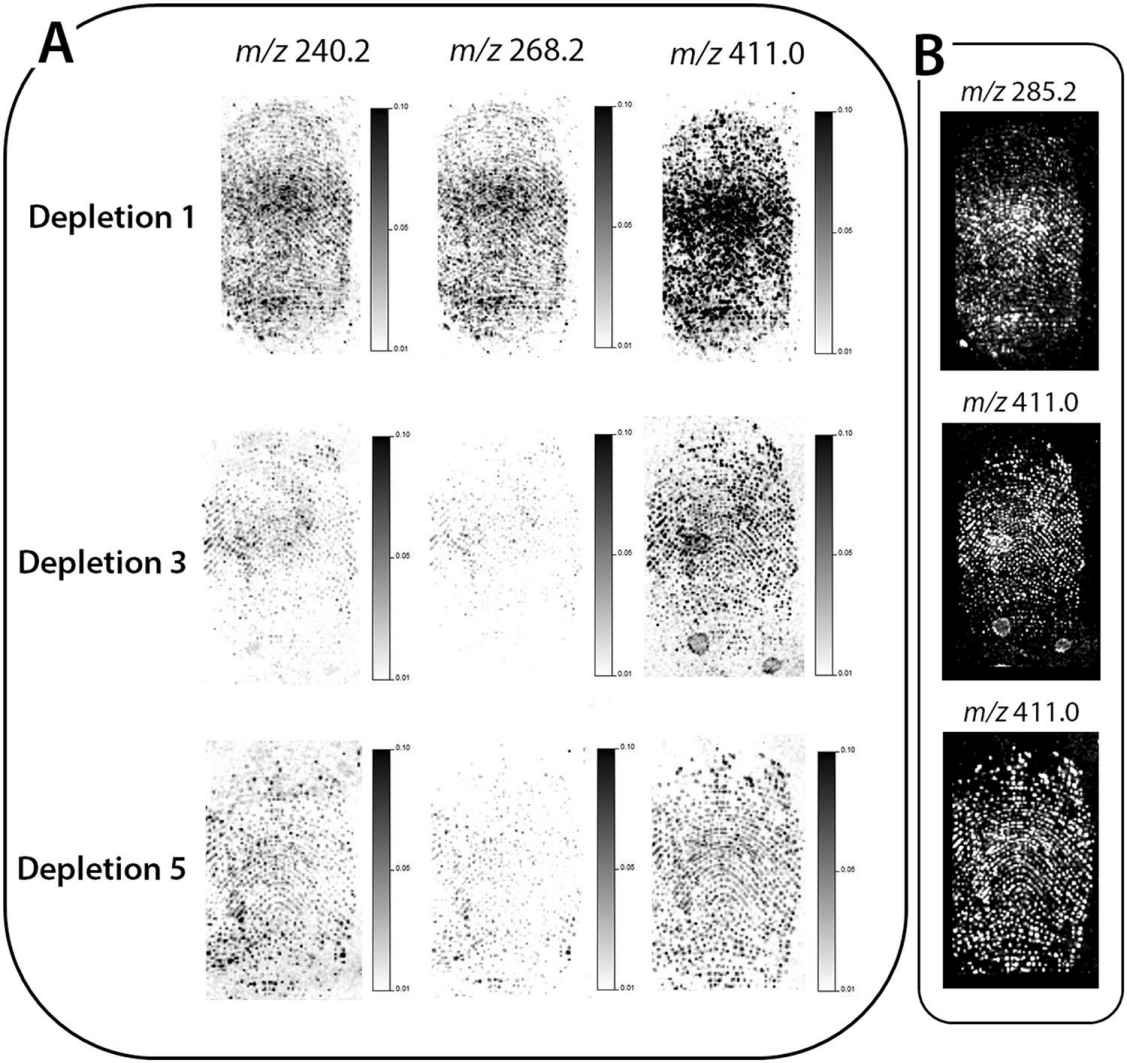
MALDI MSI of a depletion series (1^st^, 3^rd^ and 5^th^) of latent ungroomed fingermarks, aged in a wallet for 6 weeks, including; (**A**) MALDI MS images of three ion species (*m/z* 240.2, *m/z* 268.2 and *m/z* 411.0) set to the same contrast and intensity, showing a reduction in the signal and ridge detail clarity through the depletions and (**B**) ridge detail reconstruction using the ion signal with the best ridge clarity for each fingermark, showing that grade 2 images can be obtained in each instance.

All marks were deposited onto the opaque section of the polymer banknote and the 1^st^, 3^rd^ and 5^th^ fingermark of the depletion series were submitted to analysis by MALDI-MSI. When comparing the image quality of three unidentified species (*m/z* 240.2, *m/z* 268.2 and *m/z* 411.0), there was a clear reduction in the ion signal intensity and quality of the fingermark ridges across the depletion series (Fig. 6A). Previous research on polymer banknotes by Jones *et al*.^13^ highlighted that fingermarks deposited onto polymer banknotes degraded very quickly due to the semi-porous nature of the surface. Nonetheless, selection of the most abundant ion in each of the depletion series, such as putative stearic acid [M + H]^+^ (*m/z* 285.2)^31^ when visualised at an optimal contrast and intensity, confirmed that grade 2 fingermark images could still be obtained in each instance (Fig. 6B).

Bradshaw *et al*.^31^ found that ageing fingermarks under controlled humid conditions compromised the quality of ridge detail relative to fingermarks aged for the same period of time at room temperature. As the ageing conditions in this research could not be measured; the effect of the temperature, humidity and pressure on the quality of fingermarks cannot be specified. Further research surrounding aged marks on polymer banknotes in controlled conditions would allow for more accurate information regarding the interaction of fingermarks with the banknote surface over a period of time. Furthermore, it would also be interesting to analyse a note stored in more realistic conditions, whereby the protective element of the paper envelope would be removed during the ageing process.

### Detection of exogenous substances on contaminated fingermarks

Obtaining chemical information from latent fingermarks is a key advantage of spectrometric techniques, allowing for a wide range of constituents to be targeted. These may include endogenous species, such as; glycerides, fatty acids and amino acids, but also exogenous contaminants such as cosmetics^44,45^, drugs^38^ and explosives^19^.

Throughout each of the experiments in this research, the most abundant molecular ions were selected to reconstruct fingermark images. However, it is important to note that the identification of these species is unimportant (although putative molecular assignments have been provided where possible), as the primary purpose of this research was to focus on the potential of MALDI-MSI to allow for ridge detail reconstruction of fingermarks deposited on polymer banknotes. Although the clarity of the marks will vary depending on the donor and environmental conditions^44,46^, the ability to obtain chemical information on the contents of the fingermark residue has huge forensic importance, potentially providing information on the lifestyle of an individual which can help to direct an investigation. Therefore, a set of experiments was designed in order to assess the effectiveness of MALDI-MSP and MSI in the detection of forensically interesting substances, namely cocaine, deposited onto polymer banknotes.

**Figure 7:**
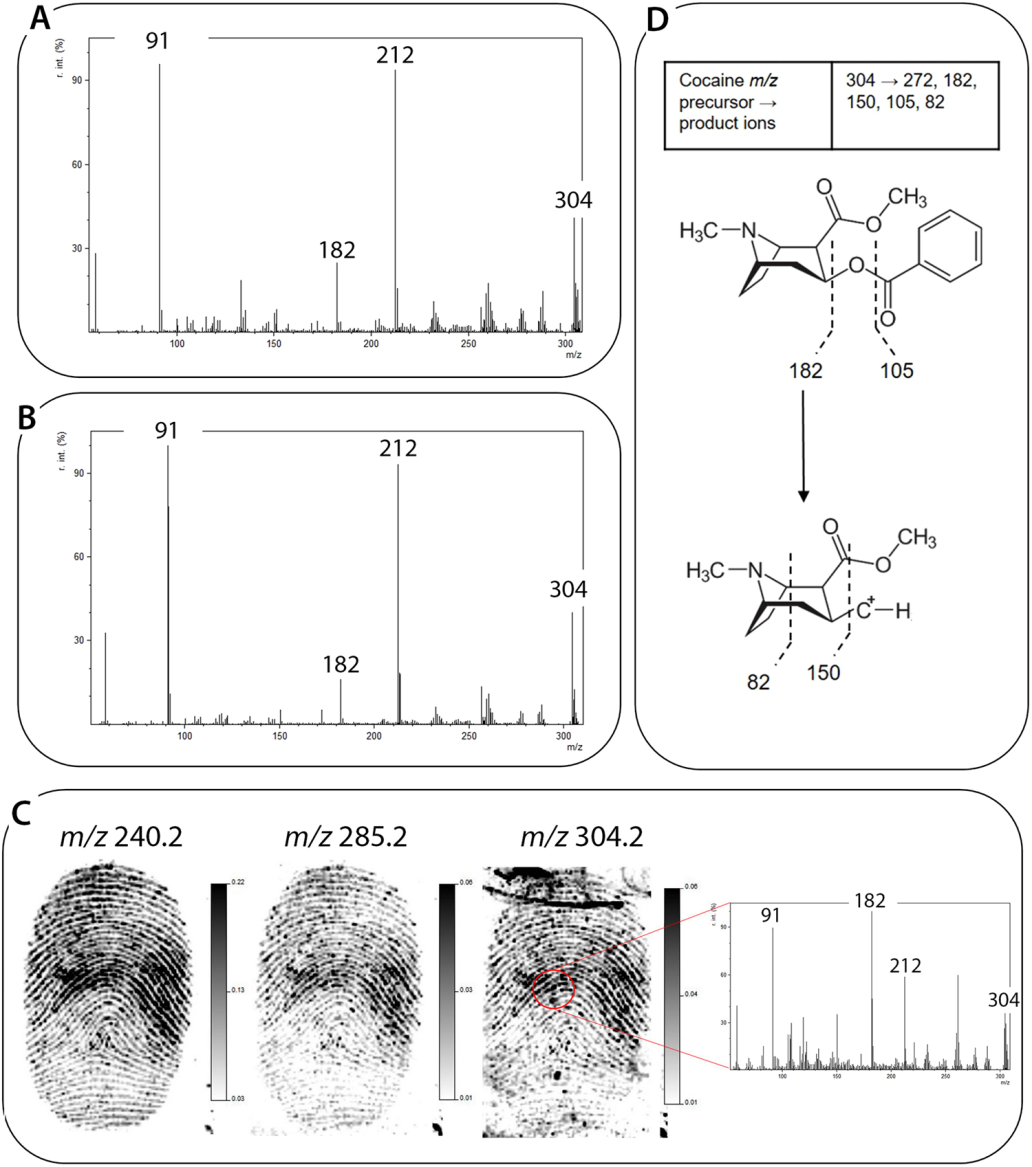
(**A**) MALDI MS/MS confirmation of 10 ng/mL cocaine spotted onto a polymer banknote, (**B**) MALDI MS/MS confirmation of 100 ng/mL cocaine spotted onto a latent ungroomed fingermark deposited onto a polymer banknote, (**C**) MALDI MS images of an unidentified species and protonated stearic acid (*m/z* 240.2 and *m/z* 285.2, respectively) and cocaine (*m/z* 304.2) of a fingermark contaminated with 100 μL of 100 ng/mL cocaine (10 ng total drug), followed by post-image MALDI MS/MS confirmation of cocaine and (**D**) the accepted fragmentation pattern of cocaine.

In an initial experiment, serial dilutions of cocaine were deposited directly onto the polymer banknote surface before being analysed by MALDI-MS and MS/MS profiling. The limit of detection (LOD) was determined based on the serial dilution which provided an MS/MS spectrum which contained product ions corresponding to that of cocaine (10 ng/mL). This corroborated previous results of Groeneveld *et al*.^38^. Ungroomed fingermarks deposited onto polymer banknotes were then spiked with three different concentrations of cocaine, starting with the value determined to be the LOD in the initial profiling experiment (10 ng/mL, 100 ng/mL and 1 µg/mL). MALDI-MSP analysis deemed the LOD of cocaine, when present within fingermark residue, to be 100 ng/mL. In both sets of experiments, MALDI-MS/MS was used to confirm the presence of cocaine through fragmentation of the precursor ion (*m/z* 304.15) to the primary product ion (*m/z* 182). Additional peaks observed in the spectra were derived from dimethylbenzylammonium (DMA) (*m/z* 304.2), with product ions observed at *m/z* 212 and *m/z* 91^47^ (Fig. 7A,B, respectively).

Finally, 100 µL of the LOD concentration observed in fingermarks (100 ng/mL) was used to contaminate a fingermark prior to deposition onto a polymer banknote and analysis by MALDI-MSI (total of 10 ng cocaine). Figure 6C shows that grade 3 fingermark images could be obtained through the selection of an unidentified species (*m/z* 240.2) and grade 2 through molecular imaging of stearic acid (*m/z* 285.2) [M + H]^+^ and cocaine (*m/z* 304.1). Confirmation of this exogenous substance was achieved by post-image MALDI-MS/MS analysis. The accepted fragmentation mechanism for cocaine has been depicted in Fig. 7D^48–50^. The total amount of drug employed here is greater than the lowest amount of cocaine detected in spiked fingermarks using MALDI MS previously^38^. However, this could still be considered a ‘low’ amount of cocaine which may be obtained from an individual handling the drug. This provides a positive indication to the sensitivity of the proposed methodology for the detection and identification of drug residues in fingermarks deposited onto polymer banknotes. The ability to differentiate between direct transfer and excretion (following drug use) would only be possible through the detection of metabolites in fingermark residue.

Cocaine has previously been shown to contaminate a significant proportion of circulated banknotes^51–53^. Drugs such as heroin, amphetamines and Δ9-tetrahydrocannabinol (THC) have also been identified on paper banknotes^52,54^. This contamination may be significantly less on the new £5 polymer banknotes due to their recent introduction (less time in circulation) and ‘cleaner’ base material. However due to the potential of background contamination, it may be necessary to conduct analysis of latent fingermarks deposited on polymer banknotes using MALDI-MSI, as opposed to profiling, as visualisation of illicit substances contained within the ridges would indicate that they are derived from the donor rather than contamination on the banknote surface (reducing the potential for false positives). The possibility of this has been demonstrated previously through discrimination between external contamination and direct deposition from a fingermark using condom lubricants^55^.

It is important to note that in all instances where cocaine was detected within fingermarks in this paper, analysis of blank regions confirmed that there was no initial contamination on the note surface prior to fingermark deposition (data not shown).

To alleviate the issue of contamination, a washing protocol was designed to reduce residual contaminants on the polymer banknotes. However, DMA was detected following MALDI MS profiling on the “cleaned” notes. This may have been introduced either in the washing protocol, in the instrument or on the banknote as a contaminant. DMA has been reported to be found in antiseptics, health care solutions and preservatives^56^, and has previously been identified as a potential contaminant in latent fingermarks^47,57^. A large scale in-depth study is required to determine background levels of different exogenous contaminants in polymer banknotes to ensure this information is taken into consideration when profiling fingermarks in forensic investigations. It would also be advantageous to assess the ability to detect metabolites of illicit substances, which has the potential to indicate drug use over handling^33,58^.

## Conclusion

The introduction of polymer banknotes into UK currency has created an issue with respect to the recovery of fingermarks from this surface in comparison to the traditional paper varieties. In a forensic scenario, currency is encountered in an array of scenarios (such as theft and money laundering); therefore it is necessary to develop methodologies which maximise fingermark visualisation. As the condition of a fingermark (whether it is depleted, aged or contaminated), location (various security features) and composition of the fingermarks will vary tremendously in each different case, a robust and reproducible methodology is imperative. In this research, MALDI-MSI has proven to be affective in addressing each of these potential issues, allowing for the analysis of minimal amounts of material in depletion series and aged marks, fingermarks deposited onto different areas of the note and following conventional development. Furthermore, MALDI-MS and MS/MS profiling has proven to be useful for the detection and confirmation of cocaine contained within the residue of fingermarks deposited onto polymer banknotes. The research presented here demonstrates the potential of MALDI-MS in providing both physical and chemical information from latent fingermarks deposited onto polymer banknotes. Ultimately, this highlights the power and sensitivity of this novel analytical approach to fingermark analysis and provides a positive indication to its potential use in forensic investigations in the future.
